# EZH2 negatively regulates PD-L1 expression in hepatocellular carcinoma

**DOI:** 10.1186/s40425-019-0784-9

**Published:** 2019-11-14

**Authors:** Gang Xiao, Li-Lian Jin, Chao-Qun Liu, Yong-Chun Wang, Ya-Ming Meng, Zhong-Guo Zhou, Jing Chen, Xing-Juan Yu, Yao-Jun Zhang, Jing Xu, Limin Zheng

**Affiliations:** 1 Collaborative Innovation Center of Cancer Medicine, State Key Laboratory of Oncology in South China, Sun Yat-sen University Cancer Center, Sun Yat-sen University, Guangzhou, 510060 People’s Republic of China; 2 Department of Biliary-Pancreatic Surgery, Sun Yat-sen Memorial Hospital, Sun Yat-sen University, Guangzhou, 510120 People’s Republic of China; 3 Guangdong Provincial Key Laboratory of Malignant Tumor Epigenetics and Gene Regulation, Sun Yat-Sen Memorial Hospital, Sun Yat-Sen University, Guangzhou, 510120 People’s Republic of China; 4 MOE Key Laboratory of Gene Function and Regulation, State Key Laboratory of Biocontrol, School of Life Sciences, Sun Yat-sen University, Guangzhou, 510275 People’s Republic of China; 5 Department of Hepatobiliary Oncology, Sun Yat-sen University Cancer Center, Sun Yat-sen University, Guangzhou, 510060 People’s Republic of China

**Keywords:** EZH2, PD-L1, Epigenetics, Immunotherapy

## Abstract

**Background:**

Accumulating studies suggest that targeting epigenetic modifications could improve the efficacy of tumor immunotherapy; however, the mechanisms underlying this phenomenon remain largely unknown. Here, we investigated the ability of the epigenetic modifier, enhancer of zeste 2 polycomb repressive complex 2 subunit (EZH2), to regulate the expression of immune checkpoint inhibitor, programmed death-1 ligand 1 (PD-L1) in hepatocellular carcinoma (HCC).

**Methods:**

Immunohistochemistry and multiplex immunofluorescence staining were performed to analyze the expression and correlation of EZH2 and PD-L1 in HCC tissues. Immunoblotting, quantitative real-time PCR, flow cytometry, chromatin immunoprecipitation, and dual-luciferase reporter gene assays were performed to evaluate the regulatory roles of EZH2 on PD-L1 expression.

**Results:**

In vitro cell experiments revealed that EZH2 negatively regulated the PD-L1 expression of hepatoma cell lines in IFNγ-dependent manner. Mechanistic studies demonstrated that EZH2 could suppress PD-L1 expression by upregulating the H3K27me3 levels on the promoters of *CD274* (encoding PD-L1) and interferon regulatory factor 1 (*IRF1*), an essential transcription factor for PD-L1 expression, without affecting the activation of the IFNγ-signal transducer and activator of transcription 1 (STAT1) pathway. Clinical samples from HCC patients with immune-activated microenvironments showed negative correlations between EZH2 and PD-L1 expression in hepatoma cells. Multivariate Cox analysis demonstrated that the combination of EZH2 and PD-L1 was an independent prognostic factor for both OS and RFS for patients with HCC.

**Conclusions:**

The epigenetic modificator EZH2 can suppress the expression of immune checkpoint inhibitor PD-L1 by directly upregulating the promoter H3K27me3 levels of *CD274* and *IRF1* in hepatoma cells, and might serve as a potential therapeutic target for combination of immunotherapy for immune-activated HCC.

## Background

Cancer immunotherapy has become an important approach to harness the immune system to fight against cancer cells [1–3]. The use of immune checkpoint blockade to induce and restore -immune activation has produced durable clinical responses in the treatment of various tumor types; however, the response rates are still low [4–8]. Increasing evidence has demonstrated the immune-regulatory properties of epigenetic modulators in some preclinical models and in patients with advanced tumors, thus suggesting a rationale for combining epigenetics and immunotherapy [9–11]. However, the underlying mechanisms by which epigenetic modifiers influence the efficacy of immunotherapy are poorly understood.

As a key component of polycomb repressor complex 2 (PRC2), enhancer of zeste 2 polycomb repressive complex 2 subunit (EZH2) mediates trimethylation on histone 3 lysine 27 (H3K27me3) and gene silencing, and is involved in various biological processes [12, 13]. Pathological activation of EZH2 histone methyltransferase (HMT) has been observed in tumor transcription programs [14], which influence cell growth [15], survival [16, 17], and metastasis [18, 19]. As a potential target for cancer therapy, the therapeutic effects of EZH2 inhibitors are generally interpreted as the consequence of direct reduction of tumor cells (TCs) [20]. Recently, studies have revealed that EZH2 can also contribute to both local and systemic antitumor immunity [21]. For example, EZH2 can affect CD8^+^ T cell-mediated anti-tumor immunity by regulating the expression of Th-1 chemokines [22, 23] or the infiltration of myeloid-derived suppressor cells (MDSCs) [24, 25]. These data suggested that EHZ2, as an important modifier, could link epigenetic regulation and immune function; however, the mechanisms underlying this phenomenon in human tumors require further explanation.

Lack of programmed death-1 ligand 1 (PD-L1) expression is an important mechanism of resistance to the anti-PD-1/PD-L1 therapies [26]. Thus, many studies have attempted to determine the biological and clinical factors involved in PD-L1 expression [27–31]. Our recent study [32] revealed that expression pattern of PD-L1 in TCs or monocytes/macrophages (Mo/Mφs) is a predictive biomarker in patients with hepatocellular carcinoma (HCC). The active immune milieu is associated with PD-L1 expression on Mφs; whereas, tumor-expressing PD-L1 may be hampered by certain cell-intrinsic modifications [32]. Given the role of EZH2 in tumor immunity, we speculated that it would have a regulatory effect on PD-L1 expression in HCC.

In the present study, we provided evidence from clinical samples and in vitro cellular experiment that hepatoma-intrinsic EZH2 represses the expression of PD-L1. The results showed that EZH2 regulates the expression of PD-L1 through the epigenetic machinery, and thus could serve as a potential therapeutic target in combination with anti-PD-L1 immunotherapy.

## Methods

### Patients and specimens

Formalin-fixed, paraffin-embedded tissue from 386 patients with pathologically confirmed hepatocellular carcinoma (HCC), who had all received resection of the tumors at the Sun Yat-sen University Cancer Center between 2006 and 2010, were enrolled as previously described [32]. All samples were anonymously coded in accordance with local ethical guidelines (as requested by the Declaration of Helsinki), with written informed consent and using a protocol approved by the Review Board of Sun Yat-sen University Cancer Center. Overall survival (OS) was defined as the interval between surgery and death or between surgery and the last observation for the surviving patients. Relapse-free survival (RFS) was defined as the interval between surgery and the first of recurrence or death, or between surgery and the last observation for patients without recurrence. Tissues were used to construct a tissue microarray (TMA) as described previously [32]. A total of 386 patients who had complete OS and RFS information were used for the survival analysis. The clinical characteristics of all the patients are summarized in Additional file 2: Table S1.

### Immunostaining and image analysis

Immunostaining and image analysis were conducted according to our previous reports [32]. In brief, TMA sections were dewaxed in xylene, rehydrated through a decreasing ethanol series, and then placed in 0.3% H_2_O_2_ to diminish the activity of endogenous peroxidase. The sections were then heated for antigen retrieval. Following incubation with rabbit anti-human EZH2 (BD Transduction Laboratories, BD Biosciences, San Jose, CA, USA), immunostaining was performed using the EnVision Detection System (DakoCytomation, Carpinteria, CA, USA) following the manufacturer’s instructions. Sections were counter-stained with hematoxylin. Image acquisition was performed using an Eclipse advanced research microscope (Nikon, Melville, NY, USA).

For multiplex immunofluorescence staining of EZH2, PD-L1 (clone: E1L3N™; Cell Signaling Technology, Danvers, MA, USA) and CD68 (DakoCytomation), Tyramide Signal Amplification (TSA) Plus Fluorescence Kits (PerkinElmer, Foster City, CA, USA) combined with immunohistochemistry (IHC) was used. To obtain multispectral images, the stained slides were scanned using the Vectra System (PerkinElmer). The definition of PD-L1 positive expression was the same as that described previously [32]. For colocalization analysis, images were acquired using a laser confocal microscope (Olympus, Essex, UK) and analyzed using FV10-ASW Viewer software (Olympus).

The expression of EZH2 was determined by nuclear EZH2 expression on tumor cells and immunohistochemical scoring of EZH2 was analyzed using the Inform software (PerkinElmer) with the modified Histo-score (H-score), which involves assessing both the intensity of staining (graded as non-staining-0, weak-1, median-2 or strong-3) and the percentage of positive cells (Additional file 1: Figure S1). The range of possible scores was from 0 to 300, quantified by H-score. The correlation of EZH2 and PD-L1 expression was analyzed by *χ*^2^ test. The cutoff value for the H-score was set at 35 with the minimum *P* value to categorize the samples into EZH2 high or low groups.

### Cells

The human hepatoma cell lines PLC/PRF/5, Huh7, and Hep3B used in this study were purchased from the American Type Culture Collection (Manassas, VA, USA). PLC/PRF/5 cells were cultured in Roswell Park Memorial Institute (RPMI) 1640 medium, and Huh7 and Hep3B cell lines were cultured in Dulbecco’s modified Eagle medium (DMEM) supplemented with 10% fetal bovine serum at 37 °C and 5% CO_2_. Hepatoma cells were treated with recombinant interferon gamma (IFNγ) (Sino Biological Inc.), DZNep (MedChemExpress, Monmouth Junction, NJ, USA), or GSK-126 (MedChemExpress) for different times and at different concentrations.

Monocytes were selected from peripheral blood mononuclear cells using anti-CD14 magnetic beads (Miltenyi Biotec, Bergisch Gladbach, Germany) as described previously [33].

### RNA interference assay

Hepatoma cells were transfected with small interfering RNAs (siRNAs) using Lipofectamine® RNAiMAX Reagent (Invitrogen, Waltham, MA, USA). Reverse transfection was performed according to the manufacturer’s instruction manual. The sequences of the siRNAs are listed in Additional file 2: Table S2.

### Flow cytometry

Cells were collected by 0.25% trypsin digestion, and incubated with Phycoerythrin (PE) conjugated PD-L1 or isotype antibodies (eBioscience, San Diego, CA, USA). The cells were then subjected to flow cytometry.

### Quantitative real-time PCR (qPCR)

Total RNA was isolated from cultured cells using TRIZOL (Invitrogen). Reverse transcription and real-time PCR were then performed using 5× All-In-One RT MasterMix (Applied Biological Materials, Richmond, Canada) and a SYBR green real-time PCR kit (Toyobo, Osaka, Japan). Relative quantification was calculated according to the comparative Ct method with normalization to the expression of *GAPDH* (encoding glyceraldehyde-3-phosphate dehydrogenase). The primers used are listed in Additional file 2: Table S3.

### Immunoblotting analysis

Cells were washed in phosphate-buffered saline (PBS) and suspended in Radioimmunoprecipitation assay (RIPA) buffer (Pierce, Rockford, IL, USA). Supernatant protein concentrations were determined using a BCA protein assay kit (Pierce). Supernatant samples were resolved by 10% or 15% SDS–PAGE depending on the sizes of target proteins, transferred to Immobilon-P polyvinylidene fluoride (PVDF) membranes (Millipore, Billerica, MA, USA) using electroblotting, and then probed with primary antibodies. Membranes were then incubated with horseradish peroxidase-conjugated secondary antibodies. The signals from the immunoreactive proteins were detected using the ECL reagent (Millipore). The information about the antibodies is listed in Additional file 2: Table S4.

### Dual-luciferase reporter assay

Huh7 and Hep3B cells pre-transfected with siRNAs, IFNγ, or not, were cotransfected with the pGL3-PD-L1 promoter-luc reporter or pGL3-basic control vectors. pRL-TK was used as an internal control. Cell lysates were harvested for the dual-luciferase assay, which was performed according to the manufacturer’s instructions (Promega, Madison, WI, USA). The primers used are listed in Additional file 2: Table S5.

### Construction of Hep3B-shEZH2 cells

To generate a cell line with the stable knockdown of *EZH2*, lentiviral plasmids carrying a short hairpin RNA (shRNA) targeting *EZH2* (VectorBuilder Inc., Shenandoah, TX, USA) were transfected to 293 T cells together with plasmids PMD2.G and pSPAX2 using Lipofectamine 3000 (Invitrogen). After 48 h, culture supernatants were collected, passed through 0.45-μm filters, and mixed with fresh media (1:1) and polybrene (8 μg/ml) to infect Hep3B cells. Cells infected with shEZH2 or control vectors were designated as Hep3B-shEZH2 and Hep3B-vector stable cell lines respectively, and were established using 1 μg/ml puromycin selection. The shRNA-targeted regions in *EZH2* were at nt 784–804.

#### Plasmids

To construction of PD-L1 overexpression plasmid, *EZH2*-silenced Hep3B cells were first treated with IFNγ for 24 h. Then, mRNA was extracted from these cells, and reverse transcription PCR was performed to obtain cDNA, which was used as template to amplify the coding sequence (CDS) of *CD274* (encoding PD-L1). After double restriction enzyme digestion (EcoR I/Kpn I), the *CD274* CDS was inserted into plasmid p3 × flag-CMV-14. To obtain more effective expression, the KOZAK sequence was designed into primers, which were as follows:

- Forward primer, GGCC GAATTC GCCGCCACC ATGAGGATATTTGCTGTCTTTATATTC;
- Reverse primer, CTGA GGTACC TTACGTCTCCTCCAAATGTGTATCACTTTG.

The *EZH2* and *IRF1* overexpression plasmids were purchased from VectorBuilder company (https://www.vectorbuilder.cn/).

### Chromatin immunoprecipitation ChIP

ChIP was performed by using SimpleChIP® Enzymatic Chromatin IP Kit (Cell Signaling Technology). Crosslinking was performed with 1% paraformaldehyde for 10 min. Micrococcal nuclease was added to digest the DNA to lengths of approximately 150–900 bp. The digested DNA solution was sonicated using a Qsonica Q700 sonicator (Qsonica, Newtown, CT, USA) for 1 min at an amplitude of 15%. Protein-DNA complexes were precipitated using specific antibodies against H3K27me3 (Cell Signaling Technology) and IgG control (Cell Signaling Technology). ChIP-enriched chromatin was used for RT-PCR with a SYBR green real-time PCR kit (Toyobo); the data were normalized to the input. The specific primers are listed in Additional file 2: Table S6.

### Prediction of CpG Islands on the CD274 promoter and bisulfite sequencing PCR

The sequence of the human *CD274* promoter was obtained from the EPD database (https://epd.vital-it.ch/index.php). MethPrimer software (http://www.urogene.org/methprimer/) was used to predict CpG islands and design bisulfite-specific primers for amplification and sequencing. Only one CpG island was predicted on the *CD274* promoter (− 2000 bp to + 500 bp). The bisulfite-specific primers sequences were as follows:

- Forward primer, ATTTGTTGTTTTGGGTAGAGGTG;
- Reverse primer, TAACTCTACTACCCCCTAAACCATC.

### Transcriptome profiling analysis

In current study, we used the same batch of transcriptome profiling data previously used [32], with differences in grouping. According to the status of immune activation which was defined by the expression level of PD-L1 on the infiltrated Mφs, the HCC tissues were divided into two groups: immune-activated and immune-suppressed [32] (12 cases in each group).

Gene with fold change (FC) more than two between groups and with a Student’s t tests *p* value < 0.05 was defined as a differentially expressed gene (DEG). And the expression data of all DEGs were analyzed to form heatmap by Funrich 3.1.3 software. The genes upregulated in immune-activated group were further analyzed for GO term enrichment by Funrich 3.1.3 software.

### Statistical analysis

Differences in the means for continuous variables were compared using Student’s *t* test or analysis of variance, and differences in the proportions were tested using the *χ*^*2*^ test. Kaplan–Meier estimates were calculated and compared using the log-rank test. Cox proportional hazard regression models were applied to evaluate the prognostic variables for OS and RFS. IBM SPSS (version 21.0; IBM Corp., Armonk, NY, USA) statistics software was used for all statistical analyses. All data were analyzed using two-tailed tests unless otherwise specified, and *P* <  0.05 was considered statistically significant.

## Results

### EZH2 negatively regulates the IFNγ-induced PD-L1 expression

**Fig. 1:**
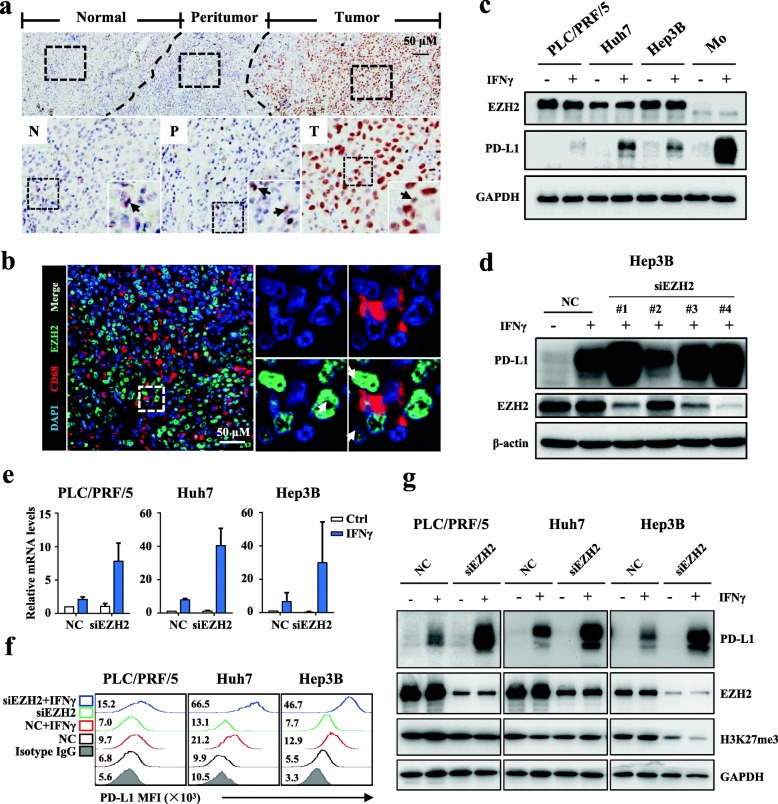
EZH2 negatively regulates the IFNγ-induced PD-L1 expression. **a** Representative IHC staining of EZH2 in HCC tissues. The black arrows indicate the expression of EZH2 on stroma cells, and the red arrows indicate the expression of EZH2 on TCs. **b** Representative pictures of multiple immunofluorescence staining showing the expression of EZH2 (green) and CD68 (red) in HCC. Scale bar, 50 μm. The white arrows indicate Mo/Mφs, and the five-pointed stars indicate TCs. **c** Immunoblotting analysis showing the expression of IFNγ-induced PD-L1 in hepatoma cells and monocytes. **d** Hep3B cells were transfected with negative control (NC) or different *EZH2*-targeted siRNAs for 48 h, and then treated with IFNγ for 24 h. Immunoblotting analyses were performed to detect the expression of EZH2 and PD-L1. β-actin was used as a loading control. **e** qPCR analysis showing that downregulation of *EZH2* promoted the mRNA expression of IFNγ-induced PD-L1 in PLC/PRF/5, Huh7, and Hep3B cells. **f** FACS staining showing that downregulation of EZH2 promoted the expression of IFNγ-induced PD-L1 in PLC/PRF/5, Huh7, and Hep3B cells. **g** Downregulation of EZH2 increased the protein level of IFNγ-induced PD-L1 in PLC/PRF/5, Huh7, and Hep3B cells. The indicated hepatoma cells were transfected with *EZH2*-targeted or NC siRNA for 48 h, and then treated with IFNγ for an additional 24 h. Immunoblotting analysis was performed to detect the protein levels of PD-L1, EZH2, and H3K27me3. GAPDH was used as a loading control

To evaluate the potential role of EZH2 in regulating PD-L1, we first investigated its expression in HCC tissues (Fig. 1a). IHC staining showed that EZH2 was highly expressed on hepatoma cells in HCC tumors compared with that on parenchyma cells in the non-tumor region. We noted that a few stroma cells were also positive for EZH2. Considering that Mo/Mφs are the major PD-L1-expressing stroma cells in HCC tumors, we performed multiplex staining to analyze EZH2 expression on Mo/Mφs. However, EHZ2 was weakly detected on Mo/Mφs in HCC tumor tissues (Fig. 1b).

Recently, we observed that over 70% of HCC tissues were weak or negative for PD-L1 expression [32]. To evaluate the effect of EZH2 on PD-L1 expression, we used different hepatoma cell lines treated with IFNγ, which is a potent PD-L1 induction factor in multiple tumors [34, 35]. As shown in Fig. 1c, hepatoma cells expressed high level of EZH2, and had only marginally increased PD-L1 expression upon IFNγ stimulation. In contrast, monocytes expressed a low level of EZH2, and showed significantly upregulated PD-L1 expression in response to IFNγ. We then performed RNAi assays to elucidate the regulatory effect of EZH2 on PD-L1. Immunoblotting assays showed that the effective *EZH2*-targeted siRNAs enhanced IFNγ-induced PD-L1 expression in hepatoma cell (Fig. 1d). Flow cytometry analyses showed that IFNγ-induced PD-L1 expression peaked at 18 to 24 h (Additional file 1: Figure S2) post stimulation. Furthermore, qPCR, immunoblotting, and flow cytometry analyses confirmed that IFNγ-induced PD-L1 expression was upregulated in a variety *EZH2*-silenced hepatoma cells (Fig. 1e-g). These data suggested that EZH2 could negatively regulate IFNγ-induced PD-L1 expression in hepatoma cells.

### EZH2-mediated H3K27me3 on the CD274 promoter to control PD-L1 expression

**Fig. 2:**
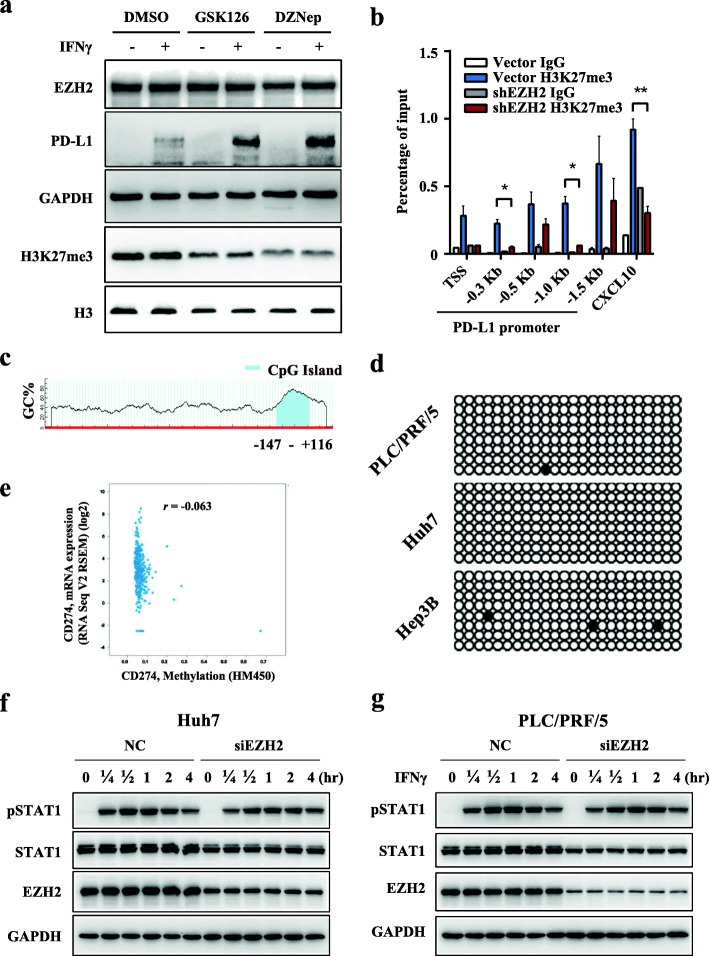
EZH2-mediated H3K27me3 on the *CD274* promoter controls PD-L1 expression. **a** Hep3B cells were pretreated with GSK126, DZNep, or DMSO for 48 h, and then treated with IFNγ for an additional 24 h. Immunoblotting was performed to detect the protein expression of PD-L1, EZH2, and H3K27me3. GAPDH and H3 were used as loading controls. **b** An H3K27me3 ChIP assay was performed in shEZH2 Hep3B and vector control cells. H3K27me3 levels at the *CD274* (PD-L1) promoter were normalized to the input. TSS, transcription start site, − 0.3, − 0.5, − 1.0, and − 1.5 kb indicate the corresponding upstream loci of the *CD274* gene TSS. CXCL10 was used as a positive control. (Mean ± S.E.M.; *n* = 3; * *P* < 0.05, ** *P* < 0.01, Wilcoxon test). **c** Diagram of the CpG island distribution on − 2000 nt to + 250 nt region of the *CD274* promoter predicted by MethPrimer website. **d** DNA methylation on the *CD274* promoter. DNA methylation at CpG sites was quantified using bisulfite sequencing. Filled circle, methylated; open circle, unmethylated. **e** DNA methylation and gene expression data for PD-L1 from TCGA HCC tissues were analyzed on the cBioportal website. The Pearson correlation coefficient (*r*) is shown. **f** and **g** Effect of downregulation of EZH2 on the IFNγ-STAT1 signaling activation. Huh7 (**f**) or PLC/PRF/5 (**g**) cells were pre-transfected with *EZH2*-targeted siRNA or NC for 48 h, and then treated with IFNγ for 0–4 h. Immunoblotting was performed to detect the levels of pSTAT1 and EZH2. STAT1 and GAPDH were used as loading controls for pSTAT1 and EZH2 respectively

EZH2-mediated H3K27me3 often leads to epigenetic silencing of target genes [36]; thus we first tested whether EZH2 could directly regulate the H3K27me3 levels on the *CD274* (the gene encoding PD-L1) promoter to suppress IFNγ-induced PD-L1 expression. A concentration gradient of DZNep (an inhibitor of all S­adenosyl­methionine (SAM)­dependent enzymes, including EZH2) [37] and GSK126 (a selective inhibitor of EZH2 methyltransferase activity) [38] were applied to reprogram the epigenetic pathways in hepatoma cells. As expected, GSK126 significantly inhibited the level of H3K27me3 without affecting EZH2 expression, while DZNep simultaneously downregulated the expression of EZH2 (Additional file 1: Figure S3a-b). Inhibiting EZH2 by GSK126 or DZNep treatment effectively increased IFNγ-induced PD-L1 expression in hepatoma cells (Fig. 2a, Additional file 1: Figure S3c-e). ChIP-qPCR analysis revealed that H3K27me3 occupancy on the promoter of *CD274* was significantly downregulated in Hep3B-shEZH2 cells compared with that of the control cells (Fig. 2b). These data suggested that H3K27me3 modification at the promoter level is involved in EZH2­mediated PD-L1 repression.

It has been reported that EZH2 could also serve as a recruitment platform for the DNA methyltransferase, DNMT1 [39]. From the MethPrimer online database, we predicted CpG islands that are enriched near the transcription start site of *CD274* [40] (Fig. 2c). However, these CpG islands are barely methylated in these hepatoma cell lines, as analyzed by bisulfite sequencing PCR (BSP) (Fig. 2d). Furthermore, The Cancer Genome Atlas (TCGA) data showed no correlation between mRNA and promoter methylation levels of *CD274* in HCC tumor tissues (Fig. 2e).

It has been reported that the upregulated expression of key molecules such as, interferon gamma receptor 1 (IFNGR1), IFNGR2, Janus kinase 1 (JAK1), and JAK2, activates the IFNγ-STAT1 signaling in response to IFNγ stimulation. We evaluated whether their expression, and the activation of IFNγ-STAT1 signaling, were influenced by EZH2. The results showed that the mRNA levels of these molecules and STAT1 phosphorylation (Fig. 2f-g) were not affected by *EZH2* silencing (Additional file 1: Figure S4a-b).

Taken together, these data demonstrated that EZH2 directly regulates the H3K27me3 levels, but not DNA methylation, of the *CD274* promoter. Moreover, activation of IFNγ-STAT1 signaling is not influenced by EZH2 expression in hepatoma cells.

### EZH2 inhibits PD-L1 expression by epigenetic silencing of IRF1 expression

**Fig. 3:**
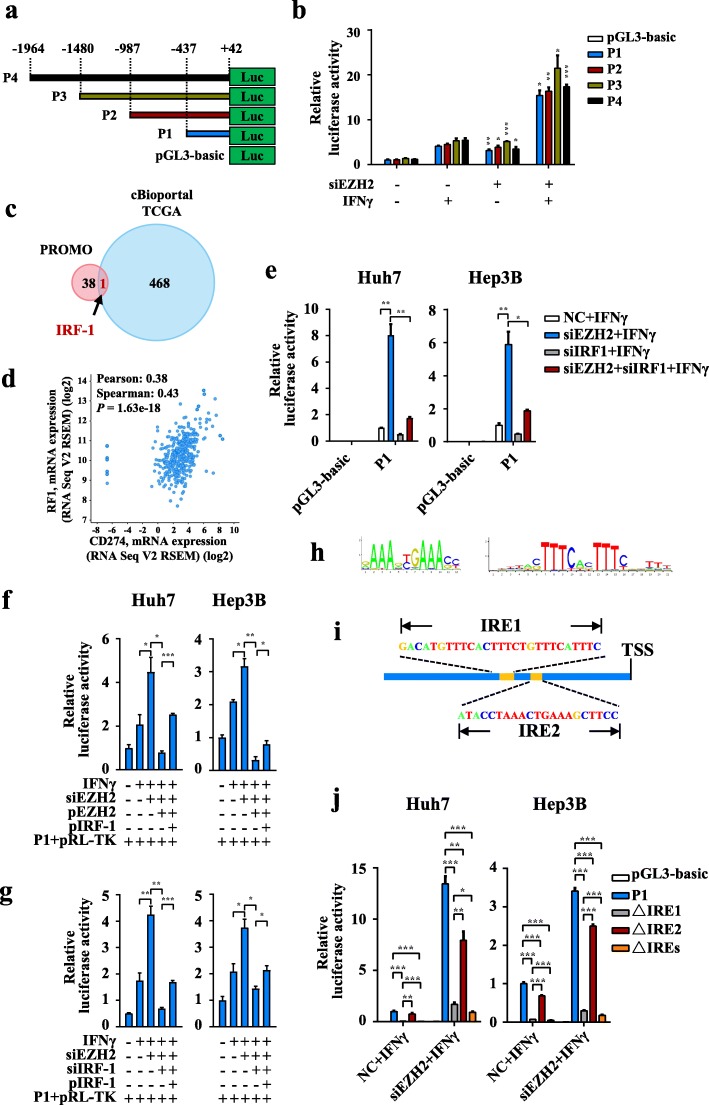
IRF1 is a potential transcription factor involved in the negative regulation of PD-L1 by EZH2. **a** Schematic diagram of a series of *CD274* (PD-L1) gene promoter luciferase reporter plasmids. **b** After transfection with *EZH2*-targeted or NC siRNA overnight, Huh7 cells were co-transfected with pGL3-basic vector or the indicated *CD274* promoter luciferase reporter gene plasmid and the pRL-TK plasmid for 48 h, and then treated with IFNγ for an additional 24-h. Luciferase activity was determined and normalized using the dual luciferase reporter system. (Mean ± S.E.M.; *n* = 3; the asterisk represents a comparison between the siEZH2 group and the corresponding control group; * *P* < 0.05, ** *P* < 0.01, *** *P* < 0.001, Wilcoxon test). **c** TFs that could potentially bind to the P1 truncated promoter were predicted using the PROMO bioinformatics software (pink circle). Genes showing the absolute values of both the Pearson and Spearman expression correlation coefficient (positively or negatively correlated) of more than 0.3 with *CD274* (PD-L1 gene) in HCC tissues (TCGA, Provisional) were analyzed on the cBioportal website (blue circle). Venn diagram showing that IRF-1 was the only candidate gene in both gene sets. **d** Scatter gram showing the mRNA expression correlation of *CD274* and *IRF1* from TCGA (HCC, Provisional). Pearson and Spearman correlation coefficients and *P* values are shown. **e** Huh7 and Hep3B cells were transfected with NC or *EZH2*-targeted, *IRF1*-targeted, or both, siRNA overnight, and then co-transfected with pGL3-basic vector or the P1 luciferase reporter gene plasmid and pRL-TK plasmid for 48 h. The cells were then treated with IFNγ for an additional 24 h. Luciferase activity was determined and normalized using the dual luciferase reporter system (Mean ± S.E.M.; *n* = 4; * *P* < 0.05, ** *P* < 0.01, Wilcoxon test). **f** After transfection with NC or *EZH2* siRNA targeting 3′-UTR, Huh7 and Hep3B cells were transfected with the indicated plasmids for 48 h, and then treated with IFNγ for 24 h. Luciferase activity was determined and normalized using the dual luciferase reporter system (Mean ± S.E.M.; *n* = 3; * *P* < 0.05, ** *P* < 0.01, Wilcoxon test). pEZH2 and pIRF-1 represent ectopic expression of EZH2 and IRF-1 respectively, and the corresponding control groups were transfected with NC siRNA and/or vector plasmids. **g** After transfection with NC or the indicated siRNA targeting 3′-UTR, Huh7 and Hep3B cells were transfected with the indicated plasmids for 48 h, and then treated with IFNγ for 24 h. Luciferase activity was determined and normalized using the dual luciferase reporter system (Mean ± S.E.M.; *n* = 3; * *P* < 0.05, ** *P* < 0.01, Wilcoxon test). pIRF-1 represent ectopic expression of IRF-1, and the corresponding control groups were transfected with NC siRNA and/or vector plasmids. **h** Sequence logo of IRF1 binding site frequency matrix of *Homo sapiens* predicted using the online software JASPAR. **i** Schematic representation of IRF1 binding sites in the CD274 P1 promoter region, as predicted by JASPAR. IRE, IRF1 response element. **j** Huh7 and Hep3B cells were transfected with NC or *EZH2*-targeted siRNA overnight, and then co-transfected with pGL3-basic vector or the indicated P1 with or without IREs sequence deletion luciferase reporter gene plasmid and pRL-TK plasmid for 48 h. The cells were then treated with IFNγ for an additional 24 h. Luciferase activity was determined and normalized using the dual luciferase reporter system (Mean ± S.E.M.; *n* = 4; NS, no significant difference; * *P* < 0.05, ** *P* < 0.01, *** *P* < 0.001, Wilcoxon test)

To further explore the regulatory effect of EZH2 on the *CD274* promoter, we constructed a promoter luciferase reporter plasmid containing different truncated versions of the *CD274* promoter without H3K27me3 modification (Fig. 3a) and examined the transcription activity of these truncated promoters using dual-luciferase reporter assays. As shown in Fig. 3b, all the designed promoters exhibited similarly enhanced luciferase activity in EZH2-silenced cells when treated with IFNγ, indicating that EZH2 might regulate *CD274* transcription by affecting the activities of certain transcription factors (TF) that bind to the P1 truncated promoter. Thirty-nine TFs were predicted to bind to the P1 promoter on the PROMO website [41, 42] and 469 genes who showed co-expression with *CD274* with correlation coefficients more than 0.3 were screened out from the cBioportal website (HCC, TCGA, Provisional) [43, 44]. The Venn diagram analysis identified IRF1 as the only potential candidate gene that met both screening criteria (Fig. 3c-d, Additional file 3: Table S7).

Next, dual-luciferase reporter assays were performed to examine the effect of IRF1 on the transcriptional activity of *CD274* promoter. The results showed that the luciferase activity of P1 promoter was enhanced by knockdown of EZH2, and this enhancement could be impaired by *IRF1* silencing (Fig. 3e). Consistent with this, ectopic expression of IRF1 partially restored the luciferase activity of P1 promoter reduced by overexpression of *EZH2* (Fig. 3f). Moreover, re-expression of *IRF1* also partly rescued the reduced luciferase activity of P1 caused by knockdown of *IRF1* in the EZH2-silenced hepatoma cells (Fig. 3g). We predicted two IRF1 response elements (IREs) on the P1 truncated promoter of *CD274* using the JASPAR database (Fig. 3h). To investigate the activities of these response elements, we constructed P1 truncated promoter luciferase reporter plasmids that were deleted for IRE1, IRE2, or both, which were named as △IRE1, △IRE2, and △IREs, respectively (Fig. 3i). Luciferase assays identified that the transcriptional activity of △IRE1, △IRE2, and △IREs were weakened as compared with wild-type P1 in Huh7 and Hep3B cells after stimulation with IFNγ (Fig. 3j). These data indicated that the transcriptional regulation of *CD274* by EZH2 is also influenced by the level of IRF1.

**Fig. 4:**
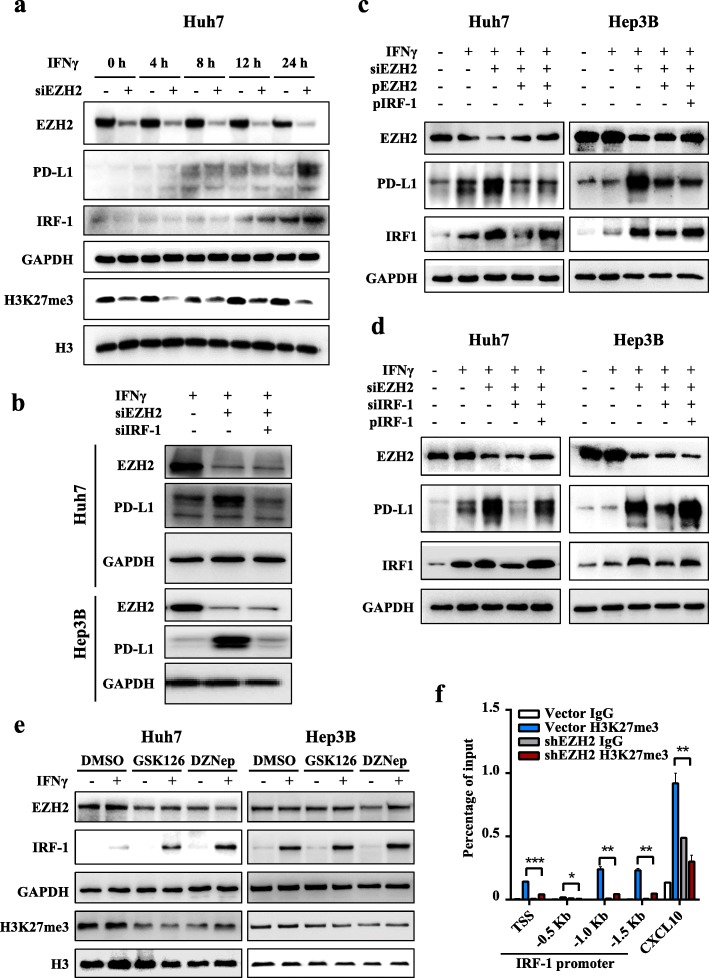
EZH2 inhibits PD-L1 transcription by inhibiting transcription factor IRF1. **a** Huh7 cells were transfected with NC or *EZH2*-targeted siRNAs for 72 h, and then treated with IFNγ for the indicated times. Immunoblotting analysis was performed to detect the levels of EZH2, IRF1, and PD-L1. GAPDH was used as a loading control. **b** Huh7 and Hep3B cells were transfected with NC or *EZH2*-targeted siRNAs, with or without *IRF1*-targeted siRNAs for 48 h, and then treated with IFNγ for 24 h. Immunoblotting was performed to detect the levels of EZH2 and PD-L1. **c** After transfection with *EZH2* siRNA targeting 3′-UTR, Huh7 and Hep3B cells were transfected with the indicated plasmids for 48 h, and then treated with IFNγ for 24 h. Immunoblotting was performed to detect the levels of EZH2, IRF-1 and PD-L1. **d** After transfection with the indicated siRNA targeting 3′-UTR, Huh7 and Hep3B cells were transfected with the indicated plasmids for 48 h, and then treated with IFNγ for 24 h. Immunoblotting was performed to detect the levels of EZH2, IRF-1 and PD-L1. In **c** and **d**, the corresponding control groups were transfected with NC siRNA or vector plasmids. pEZH2 and pIRF-1 represent ectopic expression of EZH2 and IRF-1, respectively. **e** Huh7 and PLC/PRF/5 cells were pretreated with GSK126, DZNep, or DMSO for 48 h, and then treated with IFNγ for an additional 12 h. GAPDH and H3 were used as loading controls for EZH2 and H3K27me3, respectively. **f** An H3K27me3 ChIP assay was performed in shEZH2 Hep3B and vector control cells. H3K27me3 levels on the *IRF1* gene promoter were normalized to the input. TSS, transcription start site; − 0.5 kb, − 1.0 kb, − 1.5 kb indicate the corresponding upstream locus in the *IRF1* gene TSS. CXCL10 was used as positive control (Mean ± S.E.M.; *n* = 3; * *P* < 0.05, ** *P* < 0.01, *** *P* < 0.001, Wilcoxon test)

Based on the above results, we speculated that IRF1 is downstream of EZH2 to involve in PD-L1 regulation. To examine whether IRF1 is upregulated prior to PD-L1 in EZH2-silencd cells under IFNγ treatment, we evaluated the expression dynamics of IRF1 and PD-L1. As expected, IRF1 was induced and reached its maximum level prior to that of PD-L1 (Fig. 4a, Additional file 1: Figure S5a-b). Moreover, knockdown of *IRF1* in *EZH2*-silenced hepatoma cells decreased the abundance of PD-L1 (Fig. 4b). These results suggested IRF1 should be involved in the EZH2-regulated expression of PD-L1.

Of note, we observed that the expression of IRF1 and PD-L1 were significantly reduced after the re-expression of *EZH2* in the EZH2-silenced cells; however, PD-L1 expression was only slightly increased after further ectopic expression of *IRF1* (Fig. 4c, Additional file 1: Figure S5c). Considering the epigenetic silencing of EZH2 on PD-L1, we speculated IRF1 has limited induction effect on PD-L1 expression in cells with high expression of EZH2. To further verify this, the induction effect of IRF1 on PD-L1 expression was evaluated in EZH2-silenced hepatoma cells. The results showed that knockdown of *IRF1* significantly reduced the expression of PD-L1, and this reduction was rescued by re-expression of *IRF1* (Fig. 4d, Additional file 1: Figure S5d).

We also analyzed the impact of EZH2/H3K27me3 axis inhibitors on IRF1 expression. The results showed that GSK126 and DZNep treatments promoted IFNγ-induced IRF1 expression (Fig. 4e, Additional file 1: Figure S5e). ChIP-qPCR analysis was then performed to reveal whether EZH2 could suppress the expression of *IRF1* through epigenetic machinery. The results showed H3K27me3 occupancy on the *IRF1* promoter was significantly downregulated in Hep3B-shEZH2 cells compared with that of the control (Fig. 4f). These results suggested that IRF1 expression was suppressed by the EZH2-H3K27me3 axis, which leads to inhibition of PD-L1 expression.

Taken together, these results demonstrated that IRF1 promotes the expression of PD-L1, which depends on the epigenetic modification levels of PD-L1 driven by EZH2.

### Correlation between EZH2 and PD-L1 expression in HCC tissues

**Fig. 5:**
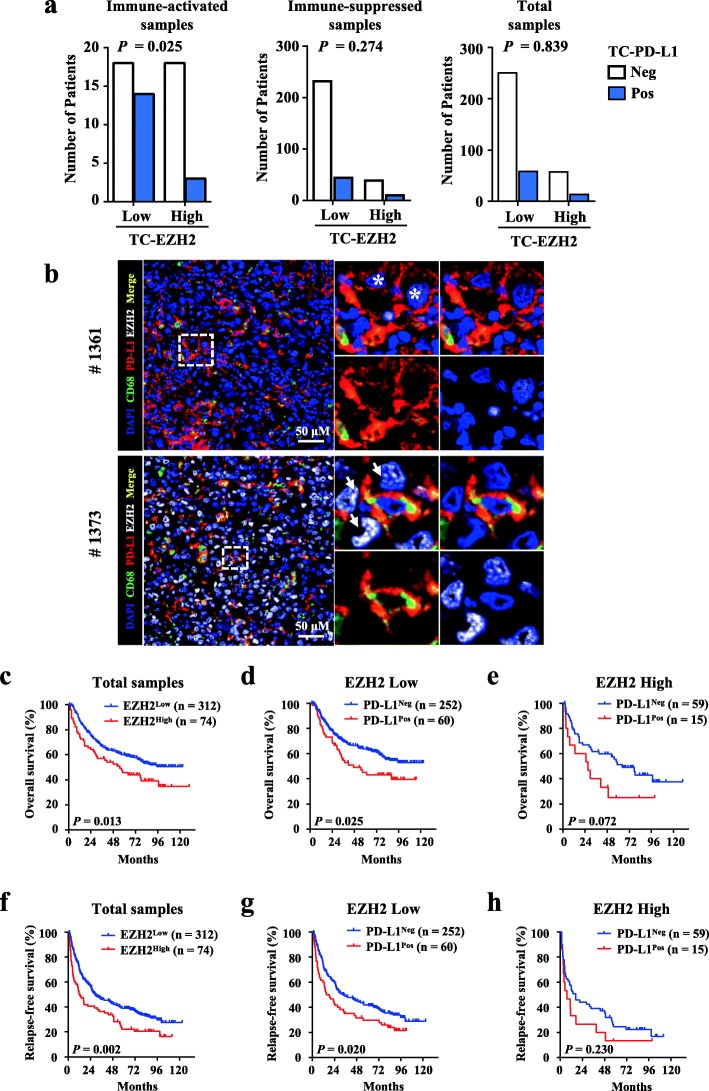
Correlation between EZH2 and PD-L1 expression in HCC tissues. **a** The expression correlation statistics of EZH2 and PD-L1 on hepatoma cells in immune-activated (left), immune-suppressed (middle), or all ungrouped (right) HCC tissues. **b** Representative images of multiple immunofluorescence staining showing the expression of EZH2 (gray) and PD-L1 (red) in HCC. Scale bar, 50 μm. #1361 and #1373 indicates the ID number of the HCC samples. The white stars and arrows indicate tumor cells expressing low or high levels of EZH2, respectively, and the five-pointed stars indicate the PD-L1^+^ Mo/Mφs with EZH2 low-expression. **c** Cumulative OS of EZH2 was calculated using the Kaplan–Meier method and analyzed using the log-rank test (*P* = 0.013). Cumulative OS of PD-L1 was calculated using the Kaplan–Meier method and analyzed using the log-rank test in patients with EZH2 low expression (**d** *P* = 0.025) and high expression (**e** *P* = 0.072). **f** Cumulative OS of EZH2 combined with PD-L1 was calculated using the Kaplan–Meier method and analyzed using the log-rank test (*P* = 0.002). Cumulative RFS of PD-L1 was calculated using the Kaplan–Meier method and analyzed using the log-rank test in patients with EZH2 low expression (**g** *P* = 0.020) and high expression (**h** *P* = 0.230). (* *P* < 0.05, *** *P* < 0.001)

The above findings indicated the effect of EZH2 in regulating IFNγ-induced PD-L1 expression. Next, we analyzed the relationship between EZH2 and PD-L1 expression in HCC tumors. Our recent study showed that Mφ-PD-L1 expression was related to the activated tumor microenvironment [32]. Transcriptome profiling and gene ontology biological analysis confirmed that Mφ-PD-L1^+^ HCC tumor samples displayed an immune-activated microenvironment and upregulated genes that were mainly involved in the IFNγ-mediated signaling pathway (Additional file 1: Figure S6a and b, Additional file 4: Table S8). Considering that IFNγ stimulation was demonstrated to be required for EZH2-mediated PD-L1 expression in the cell experiments, we analyzed the association between EZH2 and PD-L1 in samples with different microenvironments. Statistical analyses showed a significantly negative correlation between PD-L1 and EZH2 levels on TCs in immune-activated HCC tissues, but not in the total or immune-suppressed samples (Fig. 5a). Multiple immunofluorescence staining revealed that EZH2 protein was barely detected on either PD-L1^+^ Mφs or TCs (Fig. 5b). Collectively, these results suggested that EZH2 was negatively correlated with PD-L1 expression in the immune-activated HCC tumor microenvironment.

Survival analysis showed that the high levels of EZH2 in hepatoma cells indicated poor OS (*P* = 0.013) and RFS (*P* = 0.002) in HCC patients (Fig. 5c, f). Moreover, patients with low or high levels of EZH2 could be further divided into two groups by their PD-L1 expression on TCs. Kaplan–Meier curves showed that patients with PD-L1 expression on TCs had poor OS and RFS (Fig. 5d, OS *P* = 0.025; Fig. 5g, RFS *P* = 0.020) in EZH2 low-expression group but not in EZH2 high-expression group (Fig. 5e, OS *P* = 0.072; Fig. 5h, RFS *P* = 0.230). Multivariate Cox analysis demonstrated that the combination of EZH2 and PD-L1 was an independent prognostic factor for both OS (*P* = 0.01) and RFS (*P* = 0.001) in HCC patients (Table 1).

**Table 1:** Univariate and multivariate analysis of factors associated with overall survival and relapse-free survival

Variables	OS				RFS			
	Univariate	Multivariate			Univariate	Multivariate		
	*p*	HR	95% CI	*p*	*p*	HR	95% CI	*p*
Gender (female/male)	0.396				0.528			
Age,y (> 50/≤50)	0.711				0.346			
HBsAg (present/absent)	0.759				0.107			
AFP, ng/ml (> 25/≤25)	< 0.0001	1.588	1.122–2.249	0.009	0.001	1.258	0.963–1.643	0.092
Tumor size, cm (> 5/≤5)	0.011	0.905	0.645–1.271	0.565	0.040	0.928	0.706–1.219	0.590
Tumor multiplicity (multiple/solitary)	< 0.0001	1.468	1.006–2.142	0.046	< 0.0001	1.518	1.094–2.107	0.012
Vascular invasion (present/absent)	< 0.0001	3.389	2.205–5.209	< 0.0001	< 0.0001	2.257	1.547–3.293	< 0.0001
TNM stage (III + IV/I + II)	< 0.0001	1.632	1.086–2.453	0.018	< 0.0001	1.294	0.914–1.831	0.146
Differentiation (III + IV/I + II)	0.55				0.700			
Combination of EZH2 and PD-L1	0.0004	1.228	1.050–1.436	0.010	0.0002	1.254	1.100–1.430	0.001

Variables associated with overall survival or relapse-free survival by univariate analysis were adopted as covariates in multivariate analysis and entered into the equation by the forward selection based on likelihood ratio test

*Abbreviations*: *OS* Overall survival, *RFS* Relapse-free survival, *HR* Hazard ratio, *CI* Confidence interval

## Discussion

Accumulating evidence indicates that epigenetic inhibitors could improve the therapeutic efficacy of immune checkpoint blockade [21, 45]; however, the exact regulatory mechanism is not fully understood. In the present study, we reported that the epigenetic modifier EZH2 negatively regulated IFNγ-induced PD-L1 expression in hepatoma cells. Mechanistic studies demonstrated that EZH2 could suppress PD-L1 expression by upregulating the promoter H3K27me3 levels of *CD274* (encoding PD-L1) and *IRF1* without affecting activation of the IFNγ-STAT1 pathway. A negative correlation between EZH2 and PD-L1 expression on TCs was demonstrated in HCC tissues with an immune-activated microenvironment. Moreover, the combination of EZH2 and PD-L1 on TCs was an independent prognostic factor for OS and RFS in patients with HCC.

Our previous studies demonstrated the differentiated expression of PD-L1 on TCs and Mo/Mφs had opposite clinical impacts on HCC patients [32]. Transcriptome profiling analysis showed that the immune-activated microenvironment was associated with PD-L1 expression on Mo/Mφs but not TCs, indicating that the immune-induced PD-L1 expression by TCs might be regulated by certain intrinsic factors. In the present study, we found that hepatoma cells expressed high levels of EZH2, which abrogated PD-L1 upregulation by IFNγ. In contrast, Mo/Mφs had low levels of EZH2 in HCC tumors and showed significantly upregulated PD-L1 expression after IFNγ stimulation. It should be noted that the criteria for evaluation of EZH2 positive expression were ambiguous in different studies [46, 47]. We determined the expression of EZH2 by nuclear expression on tumor cells, and quantified by H-score. The cutoff value was chosen by the minimum *P* value that divided patients with diverse clinical outcomes, and also highlighted the correlation between EZH2 and PD-L1 expression. The biological and clinical significance of this cutoff value should be further validated in other cohorts. Taken together, our data revealed that EZH2 acts as an intrinsic modifier that could influence PD-L1 expression in hepatoma cells.

Our mechanistic studies showed that EZH2 epigenetically silenced IFNγ-induced PD-L1 expression by upregulation of the H3K27me3 levels on the promoters of both *CD274* and *IRF1*. Notably, EZH2 did not affect the activation of IFNγ-STAT1 signaling in hepatoma cells, as analyzed by qPCR and immunoblotting assays. This is different from other tumor models, such as in myc-driven prostate cancer, in which *EZH2* knockdown restored IFNGR1 expression and further led to activation of IFN-JAK-STAT1 signaling [48]. Recently, it has been reported that the expressions of EZH2 and PD-L1 were positively correlated in lung adenocarcinoma, and DNA methylation could be involved in regulating PD-L1 expression [49, 50]. However, our BSP analysis in hepatoma cells with low constitutive expression of PD-L1 showed almost no methylation on the *CD274* promoter regions that were predicted to be CpG islands (Fig. 2d). These data indicated that the intrinsic modifiers and extrinsic inducers for PD-L1 could be varied in different tumor models, for instance, the inflamed liver microenvironments due to chronic viral infection could have great impact on PD-L1 expression.

EZH2 inhibitor intervention experiments showed that both GSK126 and DZNep increased IFNγ-induced PD-L1 expression. ChIP-qPCR analysis showed the downregulation of EZH2 decreased the H3K27me3 levels at the *CD274* promoter. These findings suggested that EZH2 regulates PD-L1 expression in hepatoma cells partly by controlling the H3K27me3 levels on the *CD274* promoter. However, we observed that GSK126, a direct HMT inhibitor, and DZNep, an indirect HMT inhibitor, displayed certain differences in terms of promoting PD-L1 expression. These results suggested that other mechanisms might be involved in DZNep’s activity, such as proteasomal degradation of PRC2 subunits, inhibition of other methylation reactions, or reactivation of thioredoxin-binding protein 2 (TXNIP), which causes disruption of PRC2 [51].

We also found that EZH2 could control the H3K27me3 level of the *IRF1* promoter, which is prerequisite for IFNγ-induced upregulation of PD-L1 [52]. Downregulated expression of *EZH2* significantly inhibited IFNγ-induced upregulation of IRF1. Whereas, the rescue assays showed that downregulated expression of *IRF1* significantly decreased the upregulated levels of *CD274* promoter luciferase activity and protein expression caused by siEZH2 under IFNγ stimulation. In addition, in line with the results of Lee’s report [52], we identified two IRF1 response elements (IREs) on the *CD274* promoter. We further identified that IRE1 was a more effective than IRE2 in IRF1-mediated transcription of *CD274*, which might reflect the higher number of IRF1 binding sites in IRE1, as shown by sequence analysis.

In addition, we also tested other inflammatory factors, such as IL-6 and TNFα, for their induction of PD-L1 expression on hepatoma cells. However, neither IL-6 nor TNFα could induce PD-L1 expression on hepatoma cells, with or without *EZH2*-silencing (Additional file 1: Figure S7a-d). Notably, we found TNFα synergistically promoted the expression of PD-L1 induced by IFNγ (Additional file 1: Figure S7e). This might reflect its role of promoting the stability of PD-L1 [53]. We observed that compared with control cells, the degradation rates of ectopically expressed PD-L1 did not increase in *EZH2*-silenced hepatoma cells after treatment with the protein synthesis inhibitor cycloheximide (CHX), which suggested that the regulation by EZH2 of IFNγ-induced PD-L1 does not involve the protein stability of PD-L1 in hepatoma cells (Additional file 1: Figure S8).

Drugs targeting EZH2 has been shown to promote the secretion of Th1-type chemokine and subsequent local infiltration of CD8^+^ T cells in ovarian and colon cancer [22, 23]. A recent study demonstrated a promotion role of CCRK/EZH2-NF-κB/IL-6 axis in HCC by reshaping the balance of MDSCs and CD8^+^ T cells [24]. Simultaneously blockade of CCRK and PD-L1 could inhibit MDSCs accumulation and engendered CD8^+^ T cell responses in tumor tissues, resulting in eradication of HCC. In this study, we observed that downregulating the EZH2 could enhance IFNγ-induced PD-L1 expression in hepatoma cells. Survival analysis showed that patients with PD-L1 expression on TCs had poor survival in the EZH2 low-expression group. These findings collectively suggest an important role of EZH2 in reformulating the tumor immune microenvironment.

In conclusion, the present study demonstrated that the epigenetic modifier EZH2 can suppress the expression of immune checkpoint inhibitor PD-L1 by directly upregulating the promoter H3K27me3 levels of *CD274* and *IRF1* in hepatoma cells and might serve as a potential therapeutic target for combination of cancer immunotherapy for immune-activated HCC.

## Supplementary information

**Additional file 1: Figure S1.** The expression of EZH2 in HCC tissue microarray. **Figure S2.** Knockdown of *EZH2* upregulates PD-L1 expression induced by IFNγ at different time points. **Figure S3.** EZH2 inhibitors promotes the IFNγ-induced PD-L1 expression.. **Figure S4.** The IFNγ-STAT1 signaling is not regulated by EZH2. **Figure S5.** The EZH2/H3K27me3 axis regulates the expression of IRF1. **Figure S6.** Transcriptome profiling and gene ontology biological analysis of immune-suppressed and immune-activated HCC tissues. **Figure S7.** Effect of IL-6 and TNFα on the expression of PD-L1 in hepatoma cells. **Figure S8.** EZH2 did not effect the protein stability of PD-L1.

**Additional file 2: Table S1.** Patient characteristics. **Table S2.** siRNA target sequences of EZH2 and IRF1 gene. **Table S3.** Sequences of primers for quantitative real-time PCR. **Table S4.** Antibodies used in current study. **Table S5.** Sequences of primers for PD-L1 promoter luciferase reporter plasmids construction. **Table S6.** ChIP primers used in current study.

**Additional file 3: Table S7.** The genes predicted in Fig. 3c.

**Additional file 4: Table S8.** List of differentially expressed gene between immune-activated and immune-suppressed HCC tissues screened by transcriptome.

## Data Availability

The datasets used for the current study are available from the corresponding author on reasonable request.

## Abbreviations

ChIP: Chromatin immunoprecipitation
CHX: Cycloheximide
CXCL10: C-X-C motif chemokine ligand 10
EZH2: Enhancer of zeste 2
FACS: Fluorescence activated cell sorting
H3K27me3: Trimethylation on histone 3 lysine 27
HCC: Hepatocellular carcinoma
HMT: Histone methyltransferase
IFNγ: Interferon gamma
IL-6: Interleukin 6
IRE: IRF1 response element
IRF1: Interferon regulatory factor 1
MDSCs: Myeloid-derived suppressor cells
Mo/Mφs: Monocytes/macrophages
OS: Overall survival
PBS: Phosphate buffer saline
PD-1: Programmed death-1
PD-L1: Programmed death-1 ligand 1
PVDF: Polyvinylidene fluoride
qPCR: Quantitative real-time polymerase chain reaction
RFS: Relapse-free survival
RNAi: RNA interference
SDS–PAGE: Sodium dodecyl sulfate-polyacrylamide gel electrophoresis
siRNA: Small interfering RNA
TCGA: The Cancer Genome Atlas
TCs: Tumor cells
TF: Transcription factor
TNFα: Tumor necrosis factor
