# Supplementary material for: EZH2 negatively regulates PD-L1 expression in hepatocellular carcinoma
Source: J Immunother Cancer. 2019 Nov 14;7:300. doi: 10.1186/s40425-019-0784-9 (PMC6854886; doi:10.1186/s40425-019-0784-9)
Supplement: Supplementary file 1 — Additional file 1: Figure S1. The expression of EZH2 in HCC tissue microarray. Figure S2. Knockdown of EZH2 upregulates PD-L1 expression induced by IFNγ at different time points. Figure S3. EZH2 inhibitors promotes the IFNγ-induced PD-L1 expression.. Figure S4. The IFNγ-STAT1 signaling is not regulated by EZH2. Figure S5. The EZH2/H3K27me3 axis regulates the expression of IRF1. Figure S6. Transcriptome profiling and gene ontology biological analysis of immune-suppressed and immune-activated HCC tissues. Figure S7. Effect of IL-6 and TNFα on the expression of PD-L1 in hepatoma cells. Figure S8. EZH2 did not effect the protein stability of PD-L1. [file 40425_2019_784_MOESM1_ESM.pdf]

Supplementary Figure Legends

Figure S1. The expression of EZH2 in HCC tissue microarray.

Representative graph to evaluate of EZH2 protein levels by immunohistochemistry using the H-score.

Figure S2. Knockdown of EZH2 upregulates PD-L1 expression induced by IFNγ at different time points.

Hep3B cells were transfected with NC or different *EZH2*-targeted siRNAs for 48 h, and then treated with IFNγ for the indicated times. FACS staining of PD-L1 was then performed.

Figure S3. EZH2 inhibitors promote the IFNγ-induced PD-L1 expression.

Huh7 or Hep3B cells were treated with concentration gradients of **a** GSK126 (0.05, 0.2, 0.5, 2, and 5 μM) or **b** DZNep (0.2, 0.5, 2, 5, and 10 μM) for 72 h, DMSO was used as control. Immunoblotting was performed to detect the levels of EZH2 and H3K27me3. GAPDH and H3 were used as loading controls for EZH2 and H3K27me3, respectively. **c** Huh7 and **d** PLC/PRF/5 cells were pretreated with GSK126, DZNep, or DMSO for 48 h, and then treated with IFNγ for an additional 24 h. Immunoblotting was performed to detect the levels of PD-L1 and H3K27me3. GAPDH and H3 were used as loading controls for EZH2 and H3K27me3, respectively. **e** The indicated cells were pretreated with GSK126, DZNep, or DMSO for 48 h, and then treated with IFNγ for an additional 24 h. FACS was performed to detect PD-L1 expression. (Mean ± S.E.M.; $n = 3$, * $P < 0.05$, ** $P < 0.01$, *** $P < 0.001$, Wilcoxon test).

Figure S4. The IFNγ-STAT1 signaling is not regulated by EZH2.

PLC/PRF/5 **a** or Huh7 **b** cells were transfected with NC or *EZH2*-targeted siRNA for 48 h, and then treated with IFNγ for 24 h. qPCR analysis was performed to detect the mRNA expression of *IFNGR1*, *IFNGR2*, *JAK1*, and *JAK2*, the relative mRNA expression levels were normalized to that of *GAPDH*.

Figure S5. The EZH2/H3K27me3 axis regulates the expression of IRF1.

a Hep3B and **b** PLC/PRF/5 cells were transfected with NC or *EZH2*-targeted siRNA for 72 h, then treatment with IFNγ for indicted time. Immunoblotting was performed to detect the expression of EZH2, IRF1, and PD-L1. GAPDH was used as a loading

control. **c** After transfection with NC or *EZH2* siRNA targeting 3′-UTR, PLC/PRF/5 cells were transfected with the indicated plasmids for 48 h, and then treated with IFNγ for 24 h. Immunoblotting was performed to detect the levels of EZH2, IRF-1 and PD-L1. The corresponding control groups were transfected with NC siRNA or vector plasmids. GAPDH was used as a loading control. pEZH2 and pIRF-1 represent ectopic expression of EZH2 and IRF-1, respectively. **d** After transfection with NC or the indicated siRNA targeting 3′-UTR, PLC/PRF/5 cells were transfected with the indicated plasmids for 48 h, and then treated with IFNγ for 24 h. Immunoblotting was performed to detect the levels of EZH2, IRF-1 and PD-L1. The corresponding control groups were transfected with NC siRNA or vector plasmids. GAPDH was used as a loading control. pIRF-1 represent ectopic expression of IRF-1. **e** PLC/PRF/5 cells were pretreated with GSK126, DZNep, or DMSO for 48 h, and then treated with IFNγ for an additional 12 h. Immunoblotting was performed to detect the levels of IRF1 and H3K27me3. GAPDH and H3 were used as loading controls for EZH2 and H3K27me3, respectively.

Figure S6. Transcriptome profiling and gene ontology biological analysis of immune-suppressed and immune-activated HCC tissues.

a Heatmap showing differentially expressed genes between immune-suppressed and immune-activated HCC tissues. Each group contains 12 samples. **b** Histogram showing the top 15 gene ontology (GO) terms of differentially upregulated genes in immune-activated HCC tissues.

Figure S7. Effect of IL-6 and TNFα on the expression of PD-L1 in hepatoma cells.

a FACS staining of PD-L1 on hepatoma cells after treatment with IL-6, TNFα, or IFNγ. **b** FACS staining of PD-L1 on Hep3B cells when treated with 0–100 ng/ml of TNFα. Hepatoma cells were transfected with NC or *EZH2*-targeted siRNA for 48 h, and then treated with IL-6 **c** or TNFα **d** for 24 h, and FACS staining of PD-L1 was performed, IFNγ-treated cells were used as positive controls. **e** The indicated cells were treated with TNFα or IFNγ, or both, for 24 h, and then immunoblotting analysis was performed to detect the protein level of PD-L1. GAPDH was used as a loading control.

Figure S8. EZH2 did not effect the protein stability of PD-L1.

Huh7 or Hep3B cells were transfected with NC or *EZH2*-targeted siRNA overnight,

transfected with the PD-L1 plasmid for an additional 48 h, and then treated with 20 μM CHX for 0, 4, 8, or 12 h. Immunoblotting was performed to detect the levels of PD-L1 and EZH2. GAPDH was used as a loading control.

Figure S1

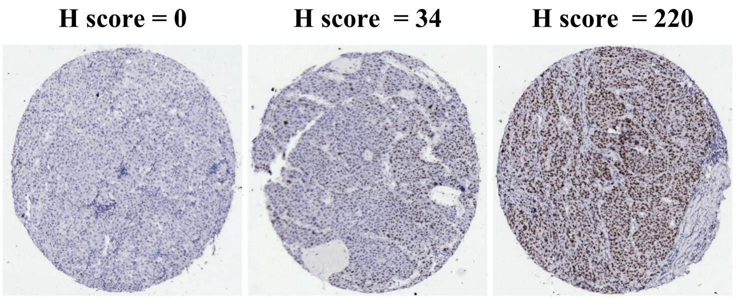

Figure S2

Hep3B

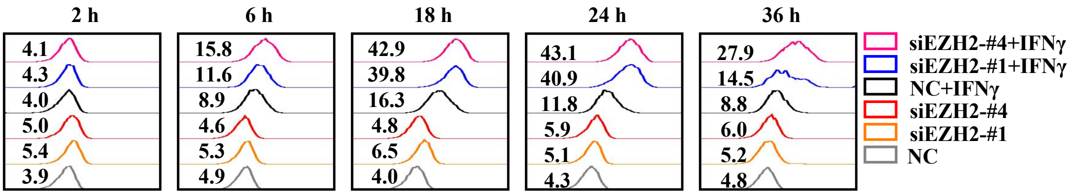

Figure S3

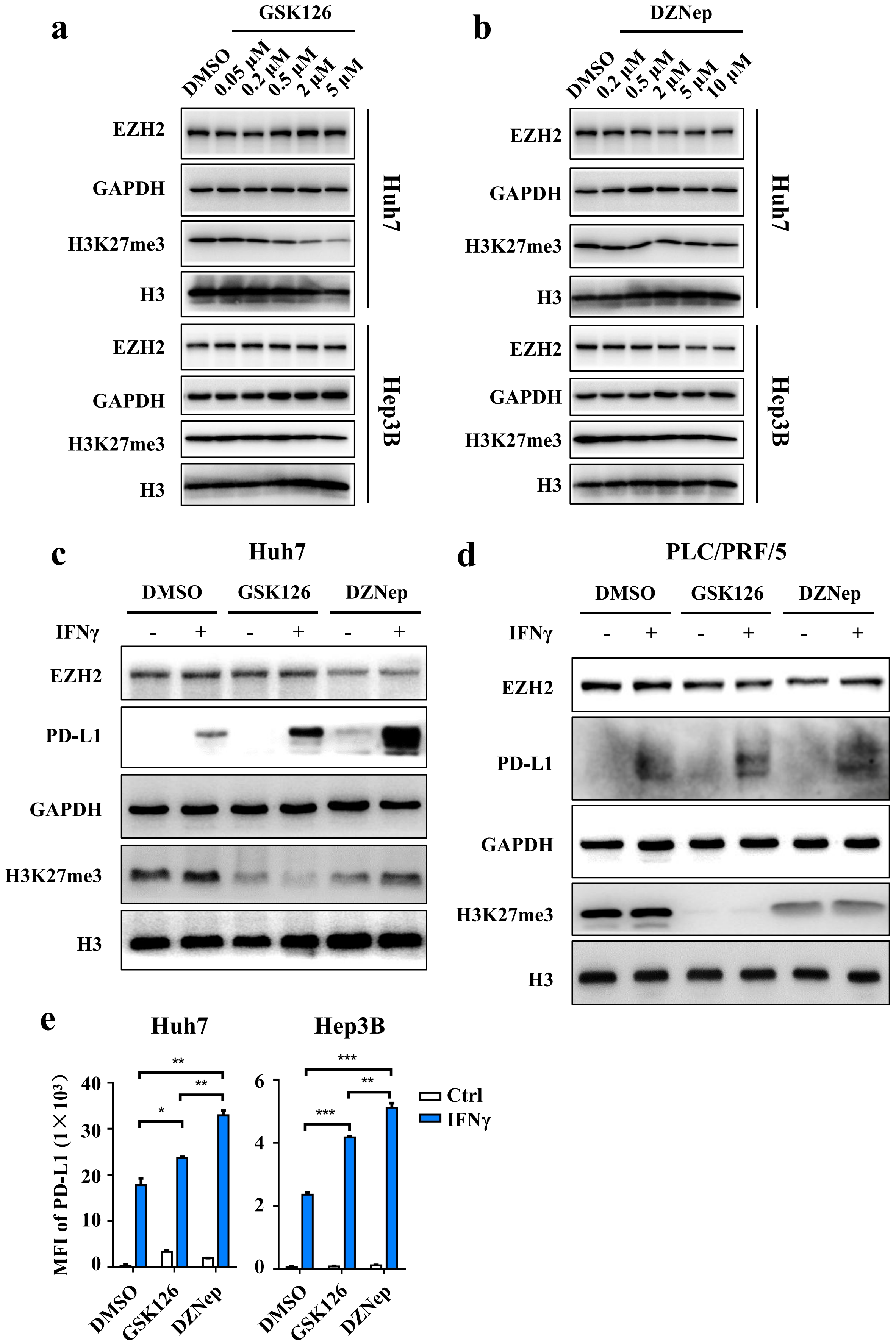

a
GSK126
DMSO
0.05 μM
0.2 μM
0.5 μM
2 μM
5 μM
EZH2
GAPDH
H3K27me3
H3
Huh7
Hep3B
b
DZNep
DMSO
0.2 μM
0.5 μM
2 μM
5 μM
10 μM
EZH2
GAPDH
H3K27me3
H3
Huh7
Hep3B
c
Huh7
DMSO
GSK126
DZNep
IFNγ
- + - + - +
EZH2
PD-L1
GAPDH
H3K27me3
H3
d
PLC/PRF/5
DMSO
GSK126
DZNep
IFNγ
- + - + - +
EZH2
PD-L1
GAPDH
H3K27me3
H3
e
Huh7
Hep3B
MFI of PD-L1 (1×10³)
Ctrl
IFNγ
DMSO
GSK126
DZNep

Figure S4

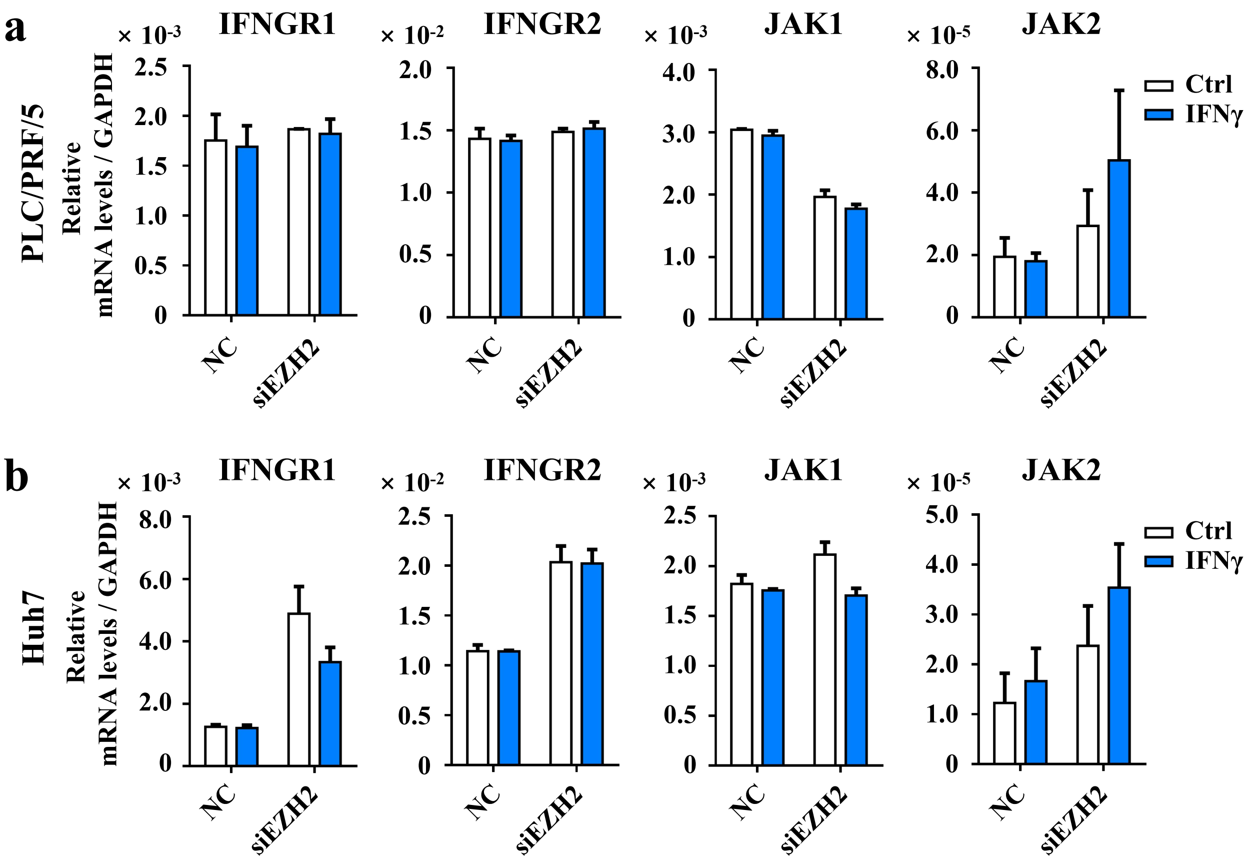
a
PLC/PRF/5
Relative mRNA levels / GAPDH
IFNGR1
× 10-3
IFNGR2
× 10-2
JAK1
× 10-3
JAK2
× 10-5
Ctrl
IFNγ
NC
siEZH2
b
Huh7
Relative mRNA levels / GAPDH
IFNGR1
× 10-3
IFNGR2
× 10-2
JAK1
× 10-3
JAK2
× 10-5
Ctrl
IFNγ
NC
siEZH2

Figure S5

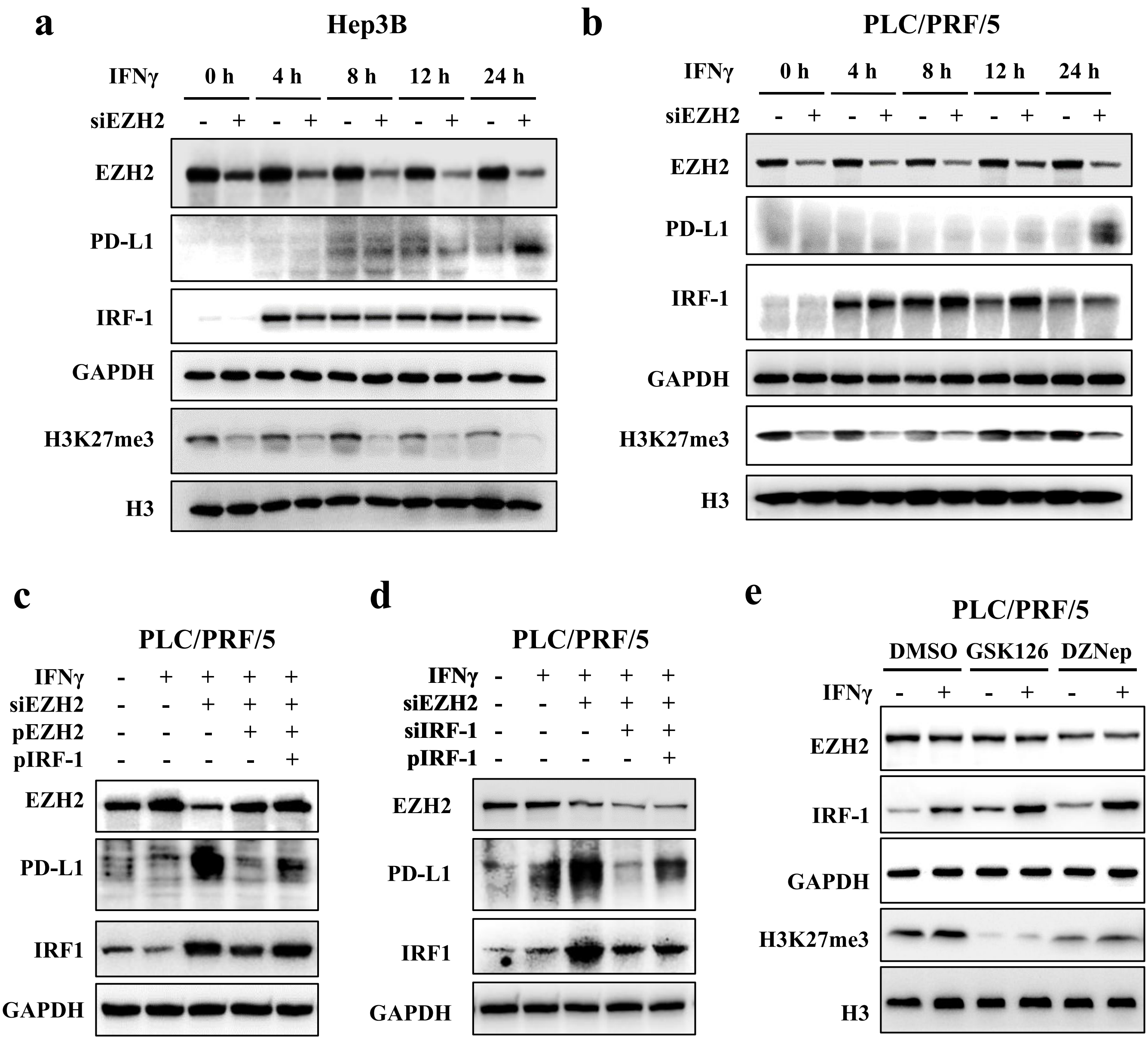
a
Hep3B
IFNγ
0 h
4 h
8 h
12 h
24 h
siEZH2
EZH2
PD-L1
IRF-1
GAPDH
H3K27me3
H3
b
PLC/PRF/5
IFNγ
0 h
4 h
8 h
12 h
24 h
siEZH2
EZH2
PD-L1
IRF-1
GAPDH
H3K27me3
H3
c
PLC/PRF/5
IFNγ
siEZH2
pEZH2
pIRF-1
EZH2
PD-L1
IRF1
GAPDH
d
PLC/PRF/5
IFNγ
siEZH2
siIRF-1
pIRF-1
EZH2
PD-L1
IRF1
GAPDH
e
PLC/PRF/5
DMSO
GSK126
DZNep
IFNγ
EZH2
IRF-1
GAPDH
H3K27me3
H3

Figure S6

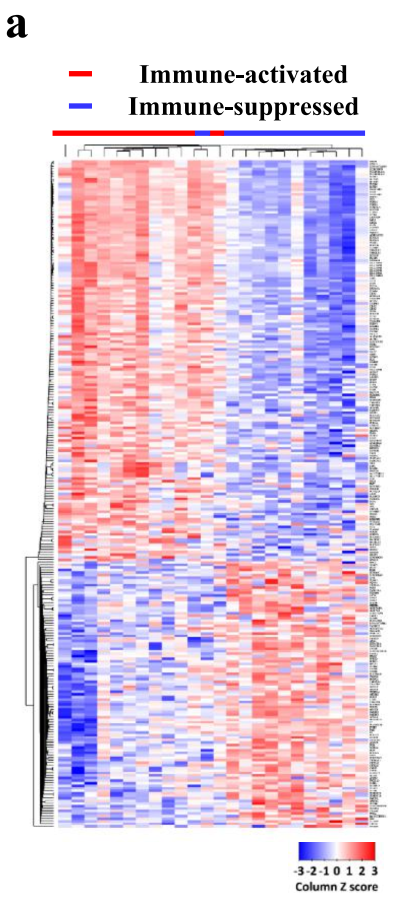
a
Immune-activated
Immune-suppressed
-3 -2 -1 0 1 2 3
Column Z score

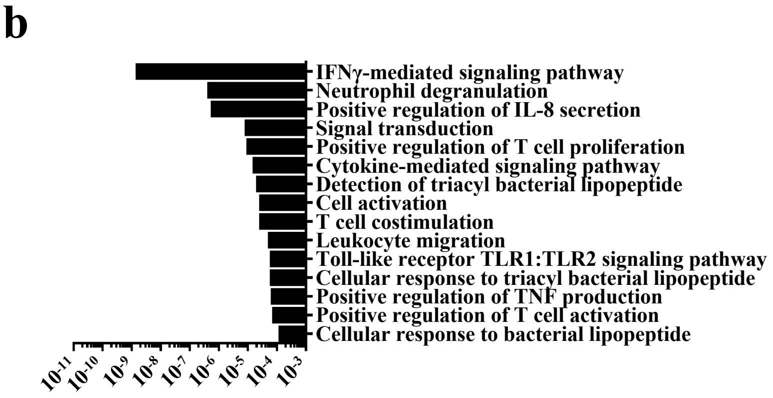
b
IFNγ-mediated signaling pathway
Neutrophil degranulation
Positive regulation of IL-8 secretion
Signal transduction
Positive regulation of T cell proliferation
Cytokine-mediated signaling pathway
Detection of triacyl bacterial lipopeptide
Cell activation
T cell costimulation
Leukocyte migration
Toll-like receptor TLR1:TLR2 signaling pathway
Cellular response to triacyl bacterial lipopeptide
Positive regulation of TNF production
Positive regulation of T cell activation
Cellular response to bacterial lipopeptide
10-11
10-10
10-9
10-8
10-7
10-6
10-5
10-4
10-3

Figure S7

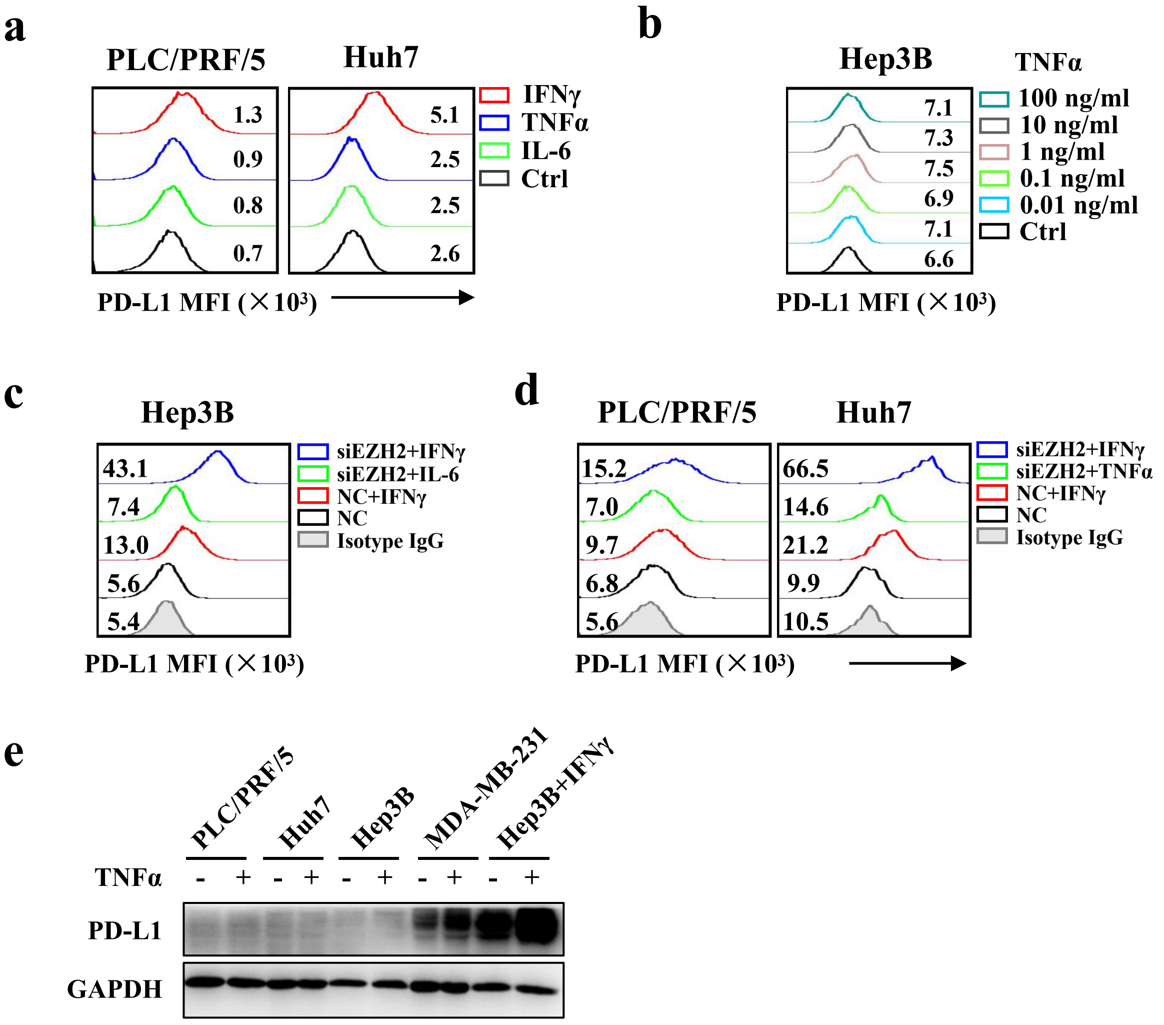

a
PLC/PRF/5
Huh7
1.3
0.9
0.8
0.7
5.1
2.5
2.5
2.6
IFNγ
TNFα
IL-6
Ctrl
PD-L1 MFI (×10³)
b
Hep3B
TNFα
7.1
7.3
7.5
6.9
7.1
6.6
100 ng/ml
10 ng/ml
1 ng/ml
0.1 ng/ml
0.01 ng/ml
Ctrl
PD-L1 MFI (×10³)
c
Hep3B
43.1
7.4
13.0
5.6
5.4
siEZH2+IFNγ
siEZH2+IL-6
NC+IFNγ
NC
Isotype IgG
PD-L1 MFI (×10³)
d
PLC/PRF/5
Huh7
15.2
7.0
9.7
6.8
5.6
66.5
14.6
21.2
9.9
10.5
siEZH2+IFNγ
siEZH2+TNFα
NC+IFNγ
NC
Isotype IgG
PD-L1 MFI (×10³)
e
PLC/PRF/5
Huh7
Hep3B
MDA-MB-231
Hep3B+IFNγ
TNFα
- + - + - + - + - +
PD-L1
GAPDH

Figure S8

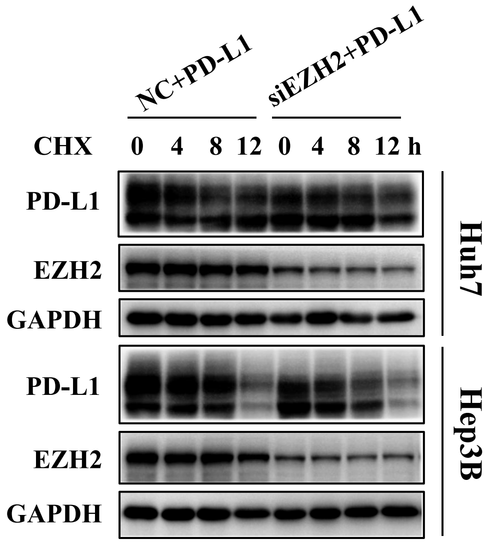
NC+PD-L1
siEZH2+PD-L1
CHX
0
4
8
12
0
4
8
12
h
PD-L1
EZH2
GAPDH
PD-L1
EZH2
GAPDH
Huh7
Hep3B
