# Supplementary material for: EZH2 negatively regulates PD-L1 expression in hepatocellular carcinoma
Source: J Immunother Cancer. 2019 Nov 14;7:300. doi: 10.1186/s40425-019-0784-9 (PMC6854886; doi:10.1186/s40425-019-0784-9)
Supplement: Supplementary file 2 — Additional file 2: Table S1. Patient characteristics. Table S2. siRNA target sequences of EZH2 and IRF1 gene. Table S3. Sequences of primers for quantitative real-time PCR. Table S4. Antibodies used in current study. Table S5. Sequences of primers for PD-L1 promoter luciferase reporter plasmids construction. Table S6. ChIP primers used in current study. [file 40425_2019_784_MOESM2_ESM.doc]

**Table S1 Patient characteristics**

| Variables | Results |
| --- | --- |
| No. Of patients | 386 |
| Age, y (median, range) | 50, 13-78 |
| Sex (male/female) | 339/47 (87.8/12.2) |
| AFP, ng/ml (median, range) | 161.1 (0-121000) |
| Tumor size, cm (median, range) | 6.0 (1.3-22.0) |
| HBsAg (present/absent) | 348/32 (91.5/8.4) |
| Tumor multiplicity (solitary/multiple) | 286/91 (74.0/23.5) |
| Vascular invasion (present/absent) | 337/39 (87.3/10.1) |
| TNM stage (III+IV/I+II) | 250/127 (64.7/32.9) |
| Differentiation (III+IV/I+II) | 226/144 (58.5/37.3) |

NOTE. Data in parentheses are percentages.

Abbreviations: HBsAg, hepatitis B surface antigen

**Table S2 siRNA target sequences of EZH2 and IRF1 gene**

| siRNA | Target sequences |
| --- | --- |
| siEZH2-1 | GAGGTTCAGACGAGCTGAT |
| siEZH2-2 | AGACTCTGAATGCAGTTGC |
| siEZH2-3 | CCAACACAAGTCATCCCATTA |
| siEZH2-4 | CCCAACATAGATGGACCAAAT |
| siEZH2-3’UTR | GAAACAGCTGCCTTAGCTT |
| siIRF1 | GCGTGTCTTCACAGATCTG |
| siIRF1-3’UTR | CAGAGGTGTACACTAACAT |

**Table S3 Sequences of primers for quantitative real-time PCR**

| Gene | Forward primer (5’-3’) | Reverse primer (5’-3’) |
| --- | --- | --- |
| IFNGR1 | GTGTGAGCAGGGCTGAGAT | TCCCAATATACGATAGGGTTCA |
| IFNGR2 | TGACAATGCCTTGGTTTCAA | ATCAGCGATGTCAAAGGGAG |
| JAK1 | GATGACAAGATGTCCCTCCG | GAATGACGCCACACTGACTG |
| JAK2 | CAGGCAACAGGAACAAGATG | CCATTCCCATGCAGAGTCTT |
| PD-L1 | TGTCAGTGCTACACCAAGGC | ACAGCTGAATTGGTCATCCC |

**Table S4 Antibodies used in current study**

| Target | Company | Application |
| --- | --- | --- |
| IRF1 | cell Signaling Technology | WB |
| Normal Rabbit IgG | cell Signaling Technology | ChIP |
| H3K27me3 | cell Signaling Technology | WB, ChIP |
| H3 | cell Signaling Technology | WB |
| GAPDH | cell Signaling Technology | WB |
| β-actin | cell Signaling Technology | WB |
| STAT1 | cell Signaling Technology | WB |
| p-STAT1 | cell Signaling Technology | WB |
| EZH2 | cell Signaling Technology | WB |
| PD-L1 | cell Signaling Technology | WB,IHC |
| EZH2 | BD Biosciences | IHC |
| CD68 | DAKO | IHC |
| PE-labeled PD-L1 | eBioscience | FACS |
| Mouse IgG1 K Isotype Control PE | eBioscience | FACS |
| Dako REAL EnVision Detection System | DAKO | IHC |
| TSA Plus Fluorescence Kits | PerkinElmer | IHC |

**Table S5 Sequences of primers for PD-L1 promoter luciferase reporter plasmids construction**

| Plasmids | Forward primer (5’-3’) | Reverse primer (5’-3’) |
| --- | --- | --- |
| P1 | GTTCAGTCACCTTGAAGAGGC | GGTACCTATCGATAGAGAAATGTTC |
| P2 | GGAAAGGCAAACAACGAAGAGTCC | GGTACCTATCGATAGAGAAATGTTC |
| P3 | CAGAGGGCATTGCAGATAGTAG | GGTACCTATCGATAGAGAAATGTTC |
| P4 | CACTGGTACCCCGGGTAGTTGATCAATTGTATGGG | TATACTCGAGAGAAGCGCGGCTGGTGCGGAGCCTC |
| △IRE1 | TATACACAGCTTTATTCCTAG | CATGTCAGTCCAGTTTTCTTG |
| △IRE2 | CTTCCGCCGATTTCACCGAA | GGTATCTAGTGTTGGTGTCC |

**Table S6 ChIP primers used in current study**

| ChIP Site | Forward primer (5’-3’) | Reverse primer (5’-3’) |
| --- | --- | --- |
| CXCL10 | GGAATGTCTCAGAAAACGTGGGGC | ACCTTCGAGTCTGCAACATGGGA |
| PD-L1_TSS | CTGGATTTGCTGCCTTGGGCAG | GAAGCTGCGCAGAACTGG |
| PD-L1_-0.3 kb | TGCGTTCAGATGTTGGCTTG | CATGTCAGTCCAGTTTTCTTG |
| PD-L1_-0.5 kb | AGGAAGTCACAGAATCCACG | AGCAGACCCATATGGCTTTG |
| PD-L1_-1.0 kb | ACCAATGCAAGGGCTATCTC | TGGACTCTTCGTTGTTTGCC |
| PD-L1_-1.5 kb | ACCTCAAGAGTCATGATGAACTAGC | GATCTACTATCTGCAATGCCCTCTG |
| IRF1_TSS | AAGGGTTAGCGTCCTGGTCTTAG | CCATTCTACGCCTTCCTGAC |
| IRF1_-0.5 kb | CCCTTCGCCGCTAGCTCTA | TGCGTGCCGTCATTTCG |
| IRF1_-1.0 kb | CAGCCGTCTGGGCTTCTC | CCCCTGGCTGGCTTTAGG |
| IRF1_-1.5 kb | GGTCACAGCACTCAGATTGCA | CCCTCCCGTAGAGGAAATGC |

**Supplementary table 7 The genes predicted in figure 3C**

| PROMO | TFs that could potentially bind to P1 promoter |
| --- | --- |
|  | JUN GATA1 TBP STAT5A NF1 FOXP3 HNF1B HOXD9 HOXD10 PGR NR3C1 CEBPB POU2F1 STAT4 ELK1 MYB E2F1 STAT1 IRF1 RXRA NFATC2 PAX5 TP53 GCFC2 PPARA NFKB1 SP1 NFATC1 AR XBP1 IRF2 VDR PEX5 HNF1A GATA2 ATF3 ESR1 NFIC FOXA1 |
| cBioportal | Genes co-expressed with CD274 in TCGA HCC tissues  (Both Pearson and Spearman r ≥0.3 or ≤ -0.3) |
|  | ADCY7 ADD2 AIM1 ABCD2 AKR1B1 TTC24 ANK1 ANXA1 AOAH FASLG CD200R1 RHOH BCAT1 BICD1 PRDM1 C1QB C1QC C3AR1 NLRC3 FMNL1 CALU CAMK4 CASP1 CASP5 RUNX2 RUNX3 CBLB CD2 CD3E CD3G CD247 CD5 CD6 CD8A CD80 CD86 TNFRSF8 TNFSF8 CD38 CD44 CD53 CD72 CDH11 CHN1 CCR1 CCR5 CCR8 CMKLR1 SCLT1 COL6A3 FAM171B CR1 CSF1R CSF2RB CYBB DYNC1LI2 DOCK2 DUSP2 DUSP4 ADGRE1 EVI2A EVI2B EYA3 FCGR1A FCGR1B FCGR2A FCGR3A GPX8 FOSL2 FPR1 FPR2 FPR3 FUT8 FYB FYN GBP1 GJA1 GPD2 XCR1 CXCR3 GZMA GZMK HCK HCLS1 NCKAP1L HIF1A HK3 HLA-DMB HLA-DOA HLA-DQA1 HLA-DQA2 HLA-DRA HRH1 HTR7 TNC IFI16 IFNG IL2RA IL2RB CXCR2P1 IL10 IL10RA IL12RB1 IL15 IL16 INPP5D IRF1 IRF4 ITGA4 ITGB2 ITK ITPKB JAK2 JAK3 KCNA3 KIF2A KLRC1 KLRC3 KLRD1 LAIR1 LCK LCP1 LCP2 LDHB LRMP LSP1 LY75 SH2D1A CIITA MICB CXCL9 MNDA MSR1 MYO1F NCAM1 NCBP1 NHS OSM TIGIT PDE3A PDE4B PDE4D PIK3CD PLA2G4A PLAU PLAUR PLCB2 PLEK PMAIP1 PML POU2F2 PRF1 SRGN PRKCB PRKCH PRKCQ MAP2K1 PTGER2 PTPN7 PTPRC PTPRO RAC2 RALGDS RBBP8 RFX3 RGS1 RORB RPS6KA1 RTN1 CCL4 CCL5 CCL18 SELPLG SRSF4 ST8SIA1 SLA SLC6A6 SLC8A1 SMARCA2 SNAPC1 SNTB2 SPI1 SPN S100Z STK10 SYK TAF4B TBXAS1 TGFBR1 PPP1R18 TNFRSF1B UCP2 VAV1 VCL WARS WAS WIPF1 ZAP70 ZNF215 PRDM2 LAPTM5 ST8SIA4 DTHD1 PTP4A2 SSPN KLRC4 EOMES C9ORF139 SLC25A53 DYRK2 SMARCA5 GPR65 ITGA8 GAS7 CST7 STX11 DOCK11 SIGLEC5 IQGAP1 CD84 SEC14L1P1 DOK2 STK17B GPR55 CD163 SLIT2 LPXN KCNK6 CHST2 AIM2 ARHGEF6 THEMIS2 AKAP5 SLC4A8 CYTIP KIAA0040 PHACTR2 TOX GIT2 MAFB ARHGAP25 TSPAN32 RASGRP1 RNF41 CD96 HNRNPR SRRM1 IGSF6 LILRB2 ZNF267 IKZF1 SIRPB1 SEMA4D CELF2 CXCR6 CD226 TNFSF13B ZNF460 MTHFD2 PDE10A HPSE LILRB1 FGL2 LILRB4 LILRA1 RAB31 DNAJB4 CORO1A MAP4K1 CD300A SAMD8 LDLRAD3 PVRIG LILRA6 ZBED2 ARSJ PARP8 LRRK1 NOL9 C14ORF37 SMG1P2 DOK3 GPR157 NAA15 CXORF21 PLEKHO2 HAPLN3 MIAT PDCD1LG2 AKNA FAM26F CMTM1 ZNF436 CD300LF FCRL6 DOCK8 ADAMTS12 MIR155HG SLAMF6 MB21D1 GBP4 UHRF2 ARL11 IL21R CLEC4A FERMT3 RSPH3 IRAK4 ABI3 CLEC1A TLR7 GMIP SLC15A3 TLR8 PYHIN1 MS4A4A NLRC5 SLA2 BIN2 AGAP2 IQCG SLF1 RAB23 TAGAP KBTBD8 GALNT7 CECR1 GPR174 CARD6 LOXL3 NFAM1 TNS4 RELT UBASH3A P2RY13 PGM2L1 C21ORF91 GPR85 GNG2 SASH3 MIOS JAKMIP1 BTN2A3P RAB39A BNC2 AHI1 SIDT1 LAX1 CCDC109B MARC1 FKBP14 TRIM62 MOB1A FAM102B GIMAP4 STYK1 SIRPG SAMD9L NID2 TFEC IKZF3 IKZF2 BMP2K FAM46A DOCK10 CHSY1 GPATCH2L SBNO2 KLRK1 CEP152 SAMD3 ADAP2 TRERF1 SLC9A9 C3ORF38 EPB41L3 FCHO1 KLHL18 AGTPBP1 FKBP15 DPY19L1 MPEG1 GPRIN3 CRTAM PIK3R5 SLAMF8 FAM78A SIGLEC10 NAV1 TWSG1 KLHL6 PELI2 ATP10D SOCS4 STRIP2 ERMN ODF2L RIC1 TSHZ3 FAM26E FGD2 SFMBT2 SLAMF7 FMNL3 FCGR1C GPR141 MS4A7 NLRC4 ZNF462 PARM1 MCOLN2 MXRA5 PAMR1 ANKRD44 MOXD1 IPCEF1 SNX20 PTPN22 SACS PLA2G2D GNB4 SOGA3 RCSD1 THEMIS RANBP6 SIGLEC7 DAPP1 CYTH4 PCED1B-AS1 SIT1 KIAA2026 GVINP1 ADAMDEC1 APOBEC3C OSCAR APOBEC3G ZNF683 ARHGAP30 SCIMP CLEC6A SYTL3 MYLIP C11ORF21 RNF19B TMTC2 CLECL1 ICOS GPR171 GPR132 PILRA TBX21 ZNF831 NCF1 ADGRE2 CLSPN MYO1G SAMSN1 PARVG XYLT1 MS4A6A ARHGAP9 MMP25 RGS18 CLEC7A IPPK P2RY12 BCL11B RASAL3 GBP6 |

r, expression correlation coefficient
